# Evaluating Soil-Borne Causes of Biomass Variability in Grassland by Remote and Proximal Sensing

**DOI:** 10.3390/s19204593

**Published:** 2019-10-22

**Authors:** Sebastian Vogel, Robin Gebbers, Marcel Oertel, Eckart Kramer

**Affiliations:** 1 Leibniz Institute for Agricultural Engineering and Bioeconomy, Max-Eyth-Allee 100, 14469 Potsdam, Germany; rgebbers@atb-potsdam.de (R.G.); moertel@atb-potsdam.de (M.O.); 2 Department of Landscape Management and Nature Conservation department, Eberswalde University for Sustainable Development, Schicklerstr. 5, 16225 Eberswalde, Germany; ekramer@hnee.de

**Keywords:** apparent electrical conductivity (ECa), pH, UAV, boundary-line, quantile regression, law of minimum

## Abstract

On a grassland field with sandy soils in Northeast Germany (Brandenburg), vegetation indices from multi-spectral UAV-based remote sensing were used to predict grassland biomass productivity. These data were combined with soil pH value and apparent electrical conductivity (ECa) from on-the-go proximal sensing serving as indicators for soil-borne causes of grassland biomass variation. The field internal magnitude of spatial variability and hidden correlations between the variables of investigation were analyzed by means of geostatistics and boundary-line analysis to elucidate the influence of soil pH and ECa on the spatial distribution of biomass. Biomass and pH showed high spatial variability, which necessitates high resolution data acquisition of soil and plant properties. Moreover, boundary-line analysis showed grassland biomass maxima at pH values between 5.3 and 7.2 and ECa values between 3.5 and 17.5 mS m^−1^. After calibrating ECa to soil moisture, the ECa optimum was translated to a range of optimum soil moisture from 7% to 13%. This matches well with to the plant-available water content of the predominantly sandy soil as derived from its water retention curve. These results can be used in site-specific management decisions to improve grassland biomass productivity in low-yield regions of the field due to soil acidity or texture-related water scarcity.

## 1. Introduction

Precision agriculture (PA) technologies are increasingly applied on arable land to improve resource efficiency, reduce environmental impacts, and optimize agricultural productivity (e.g., [1,2,3]). This can only be achieved by understanding and controlling within-field variability of soil and/or vegetation properties [4,5,6]. Soil and biomass mapping using on-the-go proximal and remote sensing are a time, cost, and labor effective way to investigate soil characteristics and biomass production, quantify their spatial variability, and delimit homogeneous management zones (HMZ) for variable rate applications [4,7,8]. 

In grassland research and management, the advent of PA has long lagged behind [9]. One major obstacle was the high variability of soil and crop characteristics, both within-field and between grassland plots, as well as the lack of knowledge in explaining the causes of such heterogeneity [9,10]. However, over the past decade numerous studies focused on that matter to close that knowledge gap (e.g., [11,12,13,14,15]). They monitored grassland biophysical parameters by means of optical remote sensing technologies [16,17,18,19,20], or mapped soil properties using proximal soil sensors, most of all applying geophysical methods to measure the apparent electrical conductivity (ECa) [11,13,21]. Especially soil ECa is recently being used for precision grassland management to characterize within-field variability of soil properties and identify HMZ. This is because ECa correlates to a series of yield effecting soil properties such as soil texture or moisture availability [22,23], while, on the other hand, ECa sensors are relatively easy to use and cause only minor damage to the grass sward [11,24].

However, only a few studies exist that combine soil data from proximal sensing with crop data from remote sensing (e.g., [14]). This is of interest because vegetation mapping by remote sensing may indicate spatial variation in a very efficient way but often does not tell the reasons for the observed variability. However, for an appropriate site-specific grassland management it is indispensable to understand the cause-effect relationship between soil properties and grassland productivity. Several soil properties such as pH, nutrient content, bulk density, water content, and soil texture can affect plant growth considerably. A couple of them are manageable (e.g., by fertilization) while others cannot be changed (e.g., soil texture) but have to be regarded in the decision making.

The overarching objective of this study was to investigate the relationship between soil characteristics and biomass production to identify high and low yielding regions within the field and their possible soil-borne causes. Specific aims were to: (i) Map a grassland field on-the-go for the pH value and the apparent electrical conductivity (ECa) using proximal soil sensors, (ii) quantify grassland biomass production from unmanned aerial vehicles (UAV)-based remote multi-spectral imagery, (iii) evaluate the spatial variability of pH, ECa, and grassland biomass using semivariance modelling, and (iv) investigate the correlation among pH, ECa, and grassland biomass by means of boundary-line analysis to evaluate causes of grassland biomass variability with special emphasis on the yield-limiting effect of pH and ECa.

## 2. Materials and Methods

### 2.1. Site Description

The grassland field Königsfeld is situated northwest of the city of Potsdam, in the federal state of Brandenburg (Germany; 5811800N, 365600E; ETRS89). According to the Koeppen-Geiger climate classification system, Potsdam belongs to the humid continental climate (Dfb). It has an average temperature of 9.2 °C with January and July being the coldest (−0.6 °C) and hottest (18.6 °C) month, respectively. The mean annual total precipitation is 566 mm with February being the driest (35 mm) and June the wettest (67 mm) month (German Meteorological Service [DWD], 1981–2010).

The soil cover consists mainly of Gleyic Cambisols of sandy soil textures [25]. Whereas Cambisols, as pedogenetically young soils, are associated with the glacial and periglacial origin of the soil’s parent material. The principal qualifier Gleyic derives from the vicinity to the Sacrow-Paretz Canal in the northeast and its related groundwater influence. However, during the field campaigns in 2016 and 2017, no water logging was observed at the soil surface. 

**Figure 1:**
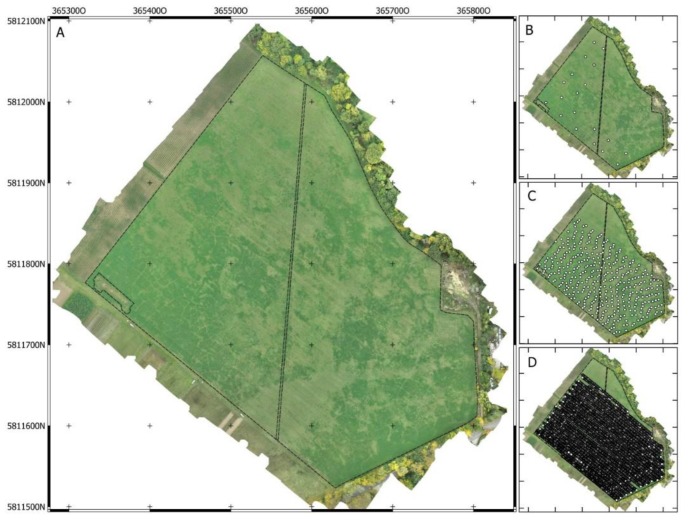
Unmanned aerial vehicles (UAV)-based orthophoto of (**A**) the Königsfeld with the locations of (**B**) biomass sampling sites, (**C**) sensor-based pH, and (**D**) apparent electrical conductivity (ECa) measurements.

The Königsfeld has a total area of 15 ha and was covered by a mixture of grass and alfalfa. The field was used as grassland since 2011, predominantly as grazing land for cattle. In the past, a fence parted the field into two halfs. Despite removal, the growing patterns of the grassland are still affected at its former location. The same applies to a former watering trough for the cattle that was situated at the western corner of the field. For that reason, both areas were disregarded in the following analyses (Figure 1A). 

### 2.2. Methods

#### 2.2.1. Proximal Soil Sensing of Grassland Soil

**Figure 2:**
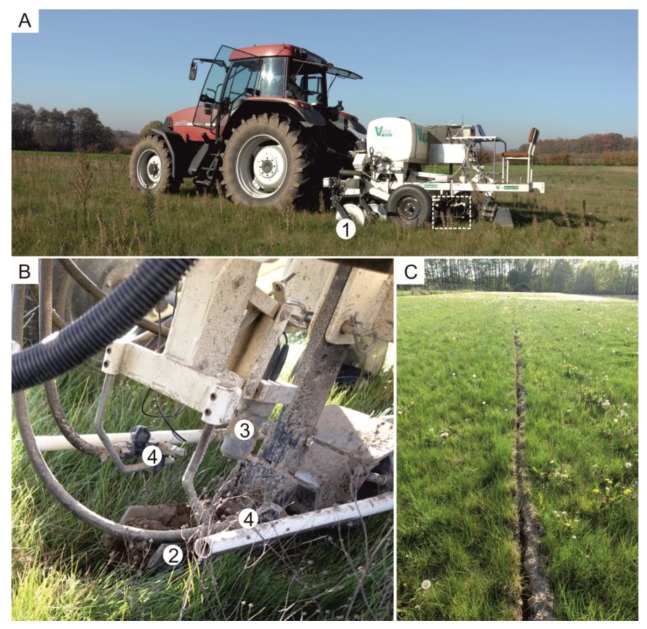
(**A**) Veris mobile sensor platform, (**B**) Veris pH-Manager, and (**C**) furrow cut by the pH sampler trough. Numbers indicate (1) rolling ECa coulter electrodes, (2) pH sampler trough, (3) pH electrodes, and (4) cleaning nozzles.

The Königsfeld was mapped in March 2017 for pH and apparent electrical conductivity (ECa) using the Mobile Sensor Platform (MSP) by Veris technologies (Salinas, KS, USA) as described by Lund et al. [26] and Schirrmann et al. [27] (Figure 2A). 

The pH value was measured on-the-go by two ion selective antimony electrodes on naturally moist soil material (Figure 2B). While driving across the field, a sampler is lowered into the soil to about 12 cm depth and a soil core flowed through the sampler’s orifice. When the soil sampler is raised out of the soil it pressed the sample against two pH electrodes for measurement. Measurements were stopped if sufficiently stable or if a maximum time of 20 s was reached. A logger recorded the raw potential data along with Global Navigation Satellite System (GNSS) coordinates. Additionally, an on-line conversion of the voltage data into pH values was conducted based on a preceding calibration with pH 4 and 7 standard solutions. After each measurement, the sampler was pushed into the soil again and the old soil core was replaced by new material that entered the sampler trough. In the meantime, the pH electrodes were cleaned with tab water from two spray nozzles to prepare them for the next measurement cycle. Typically, pH values were recorded every 10 to 12 s. GNSS coordinates were recorded when the sampler shank was raised out of the soil.

ECa was measured every second with a galvanic coupled resistivity instrument using six parallel rolling coulter electrodes (Figure 2A). This electrode configuration provided readings over two depths with a median depth of exploration of 0.12 and 0.37 m [28]. This enables the identification of significant soil textural and/or soil moisture changes between soil horizons. 

#### 2.2.2. UAV-Based Remote Sensing of Grassland Biomass

The UAV flight was performed at the end of October 2016. The UAV platform was a hexa-copter system HP-Y6 (HEXAPILOTS AHLtec, Dresden, Germany) and with a flight control software based on DJI Wookong M (DJI Innovations, Shenzhen, China). The camera was mounted on a two-axis gimbal underneath the copter. A Sony α6000 (ILCE-6000) (Sony corporation, Tokyo, Japan) RGB camera was used for image acquisition. It had a 23.5 × 15.6 mm sensor (APS-C format) with 4000 × 6000 pixels. The Sony SEL-16F28 lens had a fixed focal length of 16 mm and a maximum aperture of F2.8. The copter was navigated along a predefined route at an altitude of about 100 m. The camera produced a sequence of nadir images at fixed points along the route with an overlap of 70%. From these images an orthophoto mosaic with 10 cm resolution was generated with Agisoft PhotoScan photogrammetry software (Agisoft LLC, St. Petersburg, Russia). Before the flight, white 30 ∗ 30 cm landmarks were placed as ground control points in the field and were georeferenced with an RTK GNSS.

To estimate grassland biomass from the RGB ortho image, the following primary data and derivatives were used:

- Reflectance (R) of red, green, and blue ($R_{Red}$, $R_{Green}$, $R_{Blue}$),
- Hue,
- Saturation,
- Value,
- Normalized difference vegetation index,
  (1) $$NDVI=\frac{R_{NIR}-R_{Red}}{R_{NIR}+R_{Red}}$$
- Visible atmospheric resistant index,
  (2) $$VARI=\frac{R_{Green}-R_{Red}}{R_{Green}+R_{Red}-R_{Blue}}$$

#### 2.2.3. Reference Sampling and Laboratory Analysis

To relate the ECa data to soil texture and/or soil moisture, 25 reference soil samples were taken at locations that meet the following three requirements [29]:

- To represent extreme values of the target parameter,
- to be spatially homogeneous,
- to be well distributed throughout the area of investigation.

At each reference sampling point, five subsamples were taken with an auger from 0 to 30 cm depth within a radius of 0.5 m. The subsamples were mixed and filled in a plastic bag.

In the laboratory, the soil samples were oven-dried at 105 °C. Afterwards, the particle distribution of the fraction <2 mm was determined according to the German standard in soil science (DIN ISO 11277) by wet sieving and sedimentation after removal of organic matter with H_2_O_2_ and dispersal with 0.2 N Na_4_P_2_O_7_.

To calibrate the pH sensor data 13 reference samples were analyzed in the laboratory by the German standard method (DIN ISO 10390). The soil was dried and sieved (see above) and 10 g of it were mixed with 25 ml of a 0.01 M CaCl_2_ solution. The pH was measured with a glass electrode after 30 min.

For relating the multispectral aerial photograph on the grassland biomass, at the beginning of November 2016, aboveground biomass from 1 m² was cut at 20 points along four transects (Figure 1B). The biomass samples were weighted before and after oven-drying at 75 °C to obtain fresh and dry matter weight. 

#### 2.2.4. Data Analysis

##### Spatial Data Alignment and Visualization

Spatial data alignment and visualization was accomplished with QGIS (QGIS Geographic Information System, QGIS Development Team, Open Source Geospatial Foundation). Coordinates were transformed to a common Cartesian ETRS89 system. Image data at sampling locations and sensor measurement points were extracted by spatial queries. 

##### Analysis of Spatial Variability and Spatial Interpolation by Geostatistics

Spatial variability of the sensor data was quantified by variogram modeling [30,31,32]. The variogram can provide information about the range of spatial autocorrelation (range parameter) and the disparity between observations beyond the range of autocorrelation, which is called the sill parameter. Additionally, the nugget parameter summarizes the measurement error and sample micro-variability. For variogram modeling, firstly, the method of moments [32] was used to obtain the empirical semivariogram, which relates the average squared differences between observed values to their respective distance class (lag interval). Secondly, a theoretical semivariogram model was fitted to the empirical semivariogram. Final variogram models were established after outlier removal (see below). Calculation and visualisation was carried out with own MATLAB (The MathWorks, Inc., Natick, MA, USA) codes based on [30,31,32,33].

##### Outlier Removal by Spatial Cross-Validation

Since ECa and pH sensor measurements can be prone to error, data were checked for spatial outliers, which strongly deviate from the surrounding observations. After an initial fit of a variogram model (see above) a leave-one-out cross validation procedure was applied [32], which used the variogram model for estimating the values by kriging at each measurement location after excluding the sample value there. A linear regression was calculated between the measured and the estimated values and data points having a Cooks distance larger than 0.033 were excluded from the data set. 

##### Calibration of Sensor Data

Veris pH data: Even though the electrode readings in mV were referenced by pH 4 and 7 standard solutions, the measurements of actual soil samples need further calibration to match them with the standard laboratory method. This was necessary because the standard laboratory method extracts more protons from the soil due to the longer extraction time and because glass electrodes show higher sensitivity/responsiveness compared to antimony electrodes, which results in a trade-off between the laboratory method and the mobile sensor measurements, in particular at lower pH values. Since outlier removal (see above) reduced the number of samples that were co-located with sensor measurements, also samples which fall in a four-meter distance to sensor measurements were included in the calibration. A spatially weighted linear regression was applied which yielded a slope of 0.35, an intercept of 4, a RMSE of 0.2, and a Pearson’s r of 0.66.

RGB image: Data from the primary RGB image and derived indices were picked with the zonal statistics procedure of QGIS within a four-meter radius around the biomass sampling locations. Biomass (fresh and dry) was related to image data by linear regression. 

##### Modeling of Growth Response by Boundary-Line Analysis

Under outdoor conditions, biomass development can be restricted by various environmental factors in space and time. Consequently, the bivariate correlation between environmental factors and biomass of a whole field very often show only weak relationships and statistical distributions of non-constant variances. This is because a particular factor controls biomass development only in a subset of field observations, namely when all other growth factors are in their optimum range. This fact was first noted by Carl Sprengel in 1828 and later popularized by Justus von Liebig as the “Law of the Minimum” or “Liebig’s Law”. Consequently, for quantifying the effect of a single factor on biomass development in observational studies (uncontrolled experiments), where several factors can be limiting in different situations, the assumption of a symmetric error distribution in classical regression analyses is not valid. Instead, Webb [34] suggested to use the observable upper boundary of the point cloud in a bivariate scatter plot to describe the cause-and-effect relationship (with the limiting factor on the abscissa and the biomass or yield on the ordinate). While this early approach was mainly biologically motivated, mathematical solutions were presented later by Koenker and Bassett [35], Kaiser and Speckman [36], and Lark and Milne [37]. In particular, quantile regression is a powerful tool to detect relationships between variables when an asymmetric distribution of errors (residuals) is assumed [35]. When estimating an upper quantile (e.g., 90^th^, 95^th^, or 99^th^ percentile) of the conditional distribution of a dependent variable, quantile regression can model the effect of limiting factors by masking the effect of other unknown or unmeasured limiting factors [38,39,40]. From controlled experiments (with all factors but one being optimum) it was learned that growth-controlling factors often vary between too low, optimum, and too high [40]. This is particularly true for pH: Many crops show increasing growth depressions if pH values sink below 5.5 and rise above 7.5 while pH values around 6.5 are optimum. This effect can be modeled by a piecewise linear function with a trapezoidal shape. Fitting of trapezoidal models of growth response by quantile regression was implemented in MATLAB based on the loss function as described in Koenker and Bassett [35].

## 3. Results and Discussion 

### 3.1. Descriptive Statistics and Correlation Between Grassland Biomass and Multi-Spectral Indices

**Figure 3:**
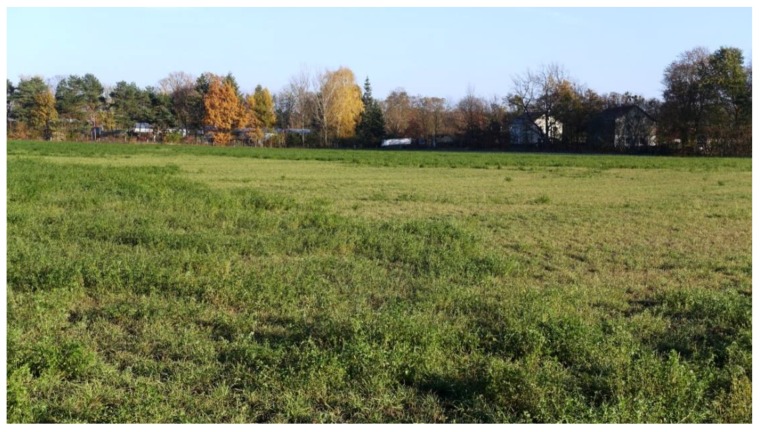
Visible small-scale variation of the grassland vegetation at Königsfeld. Alfalfa is dominant in the fore- and background while mainly graminaceous plants can be seen in the midst of the image.

Descriptive statistics of the sensor and laboratory data are shown in Table 1. During the field survey at 7 November 2016 as well as by examination of the UAV-based aerial photograph from 24 October 2016, variation in stand height and species composition was clearly visible. In areas of taller and denser vegetation, alfalfa was dominant, whereas zones of smaller and rather sparse vegetation were characterized by grasses (Figure 1A and Figure 3).

**Figure 4:**
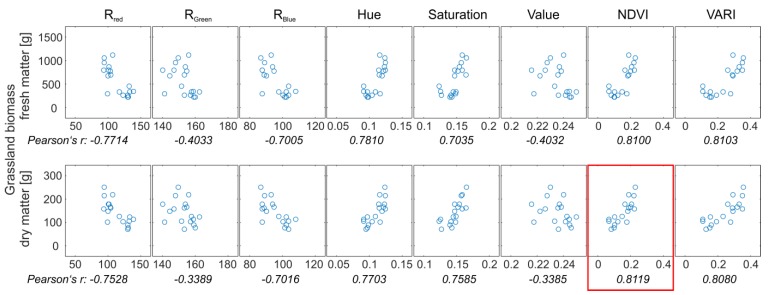
Scatterplots and correlations between fresh and dry matter weight of the grassland biomass and multi-spectral indices deduced from the UAV-based aerial photographs.

**Figure 5:**
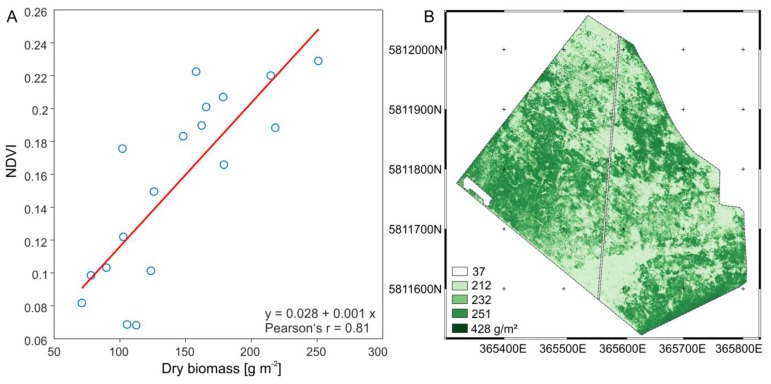
(**A**) Linear regression model of grassland biomass (dry) and normalized difference vegetation index (NDVI) and (**B**) grassland biomass estimation for the entire field.

To estimate area-wide grassland biomass of Königsfeld, multi-spectral indices derived from the UAV-based orthophoto mosaic were checked for correlation with fresh and dry matter (Figure 4). Hue, saturation, normalized difference vegetation index (NDVI) and visible atmospheric resistant index (VARI) correlated well with fresh and dry matter weight of the biomass (Figure 4). Despite the risk of insufficient estimation of high biomass due to saturation of vegetation indices at high densities [41,42] the correlation between NDVI and dry matter weight was strongest having a Pearson correlation coefficient (Pearson’s r) of 0.812. The NDVI was used to generate a regression model for the quantification of dry matter (DM) grassland biomass for the entire field (Figure 5). To derive DM, the regression model was rearranged (Equation (3)). The RMSE was 36.1 g.

(3) $$DM=\frac{\left(NDVI-0.028\right)}{0.001}$$

### 3.2. Spatial Variability of Grassland Biomass, pH, and ECa 

**Figure 6:**
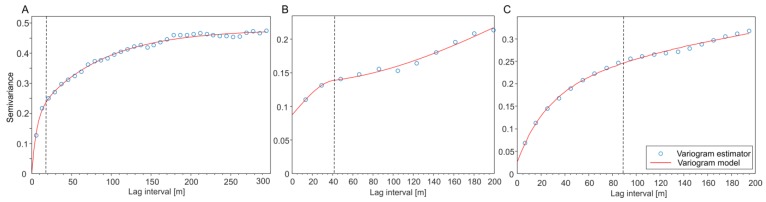
Empirical semivariograms and fitted models of (**A**) NDVI derived grassland biomass [dry matter weight, g m^−2^], (**B**) pH value, and (**C**) ECa[mS m^−1^]. Dashed vertical lines indicate the range of the first component of the nested theoretical models.

The results of spatial variability assessment of the sensor data by means of semivariance modeling are shown in Figure 6. As for the grassland biomass, pH and ECa, two nested models plus the nugget effect were fitted. That means that there were at least two spatial processes with different ranges. Variography of grassland biomass, derived from NDVI, result in a very low nugget effect as the first component of spatial correlation. This indicates a very low spatial micro-variance and a very low random error from the measurement. Two spherical models were fitted components of spatial (macro) correlation. The range of the first spherical model is about 18 m and that of the second is 245 m (Figure 6A). From the first range, it can be deduced that about half of the spatial variance of grassland biomass already occurs at distances smaller than 18 m. This corresponds to the visual impression of the UAV-based grassland biomass map (Figure 1A and Figure 3) showing pronounced small-scale disparities. 

The empirical semivariogram of the pH value also exhibits a nested structure (Figure 6B). The nugget effect is relatively high which can be attributed (a) to microvariance, which is partly to the small sensor footprint and (b) to measurement errors. The first spatial macro process was described by a spherical model with a range of 41 m indicating a high small-scale pH variance of the soil. This can be generally attributed to the high degree of soil variability in Brandenburg due to the Pleistocene and Holocene origin of its parent material and is especially amplified by decades of agricultural use [43,44,45]. Finally, the third component of spatial correlation within the pH semivariogram was described a Gaussian model showing a range of 295 m.

As with the biomass, the semivariogram model of the apparent electrical conductivity (ECa) was also characterized by a very low nugget effect (Figure 6C). Two nested exponential model components are showing ranges of 89 and 900 m.

The semivariance models have shown that most of the spatial variability of grassland biomass, pH, and ECa occurs at relatively low distances of 18, 41, and 89 m. This illustrates the necessity of small-scale data acquisition of soil and plant properties for precision agriculture applications.

### 3.3. Relationship Between ECa, pH, and Grassland Biomass

**Figure 7:**
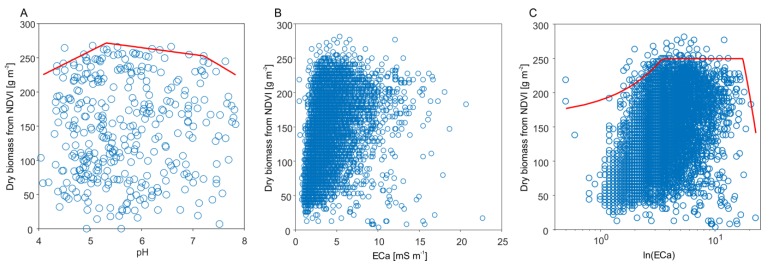
Association between pH, (**A**) ECa, (**B**) log-transformed ECa, (**C**) and grassland biomass of a alfalfa-grass mixture and modelling of their yield-limiting effect by means of quantile regression.

To investigate the relationship between sensor-based pH value, ECa data, and grassland biomass, in a first step scatterplots were created (Figure 7). At first sight, the scatterplots show no correlation between the dependent and the independent variable and, especially visible for the ECa data, a non-constant variance (Figure 7B). In contrast, the variance of ECa clearly increases with increasing grassland biomass which violates one of the key assumptions of linear regression. This is generally due to the complex nature of factors effecting biomass production. In the context of Liebig’s law of the minimum, the variability in the biomass data cannot be entirely explained by ECa and the pH value alone since many other unmeasured factors are a potentially limiting resource for plant growth [46]. Thus, instead of linear regression analyzing the correlation of the means, quantile regression was applied estimating the 95^th^ percentile of the conditional distribution of grassland biomass. This masks the effect of other unknown or unmeasured yield-limiting factors (e.g., [36,38,39]) and the limiting effect of ECa and the pH value can be visualized by means of the boundary line.

A trapezoidal boundary-line model fitted best to the 95^th^ percentile of the pH value (Figure 7A). It characterizes three stages of response of alfalfa-grass mixture productivity to soil pH [47]: A linear yield increase until a pH value of 5.3, a middle stage of yield stability consisting of a light negative slope until pH 7.2, and a linear yield decline with increasing pH values. This corresponds with several publications stating a pH optimum for alfalfa between 5.8 and 7.2. However, some of these references [48,49,50] indicate that there might be an interaction between pH and soil texture: The pH optimum shifts to a higher range on fine-textured soils and to a lower range on sandy soils. As soil texture at Königsfeld is strongly dominated by sand (86% sand, 11% silt, and 3% clay) this could be a reason for observing a lower threshold of optimum pH at 5.3. For management of the field it can be considered to raise the pH value by liming at sites with pH lower than 5.3. In contrast, lowering too high pH values (>7.2) is more difficult, in particular if the high pH is due to carbonaceous parent material. The farmer might, at least, avoid any liming in these areas and can reduce other inputs as well. 

**Figure 8:**
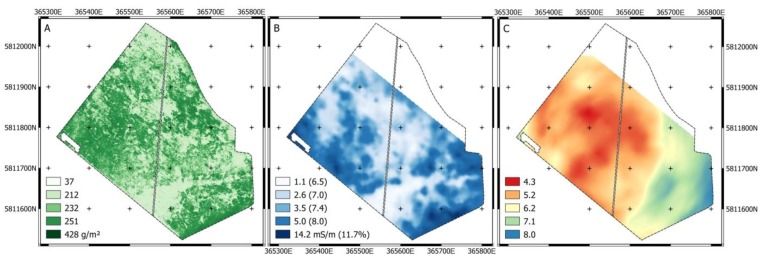
(**A**) Sensor-based estimate of dry grassland biomass, (**B**) soil ECa_deep_ with soil moisture in parentheses, and (**C**) pH value at Königsfeld.

Before applying quantile regression to ECa, the data were log-transformed (log_10_) to improve visibility of patterns in the data (Figure 8C). Additionally, for the ECa data a trapezoidal boundary-line model matched best with the 95^th^ percentile. The model can be interpreted as follows: Biomass increases until 3.5 mS m^−1^, no limits related to ECa are imposed until 17.5 mS m^−1^, and biomass decreases with increasing ECa above 17.5 mS m^−1^. In fact, by visually comparing the grassland biomass with the ECa_deep_ map, it is striking that especially low (light green) and high (dark green) values of grassland biomass coincide well with low and high ECa_deep_ values, respectively (Figure 8A,B). 

**Figure 9:**
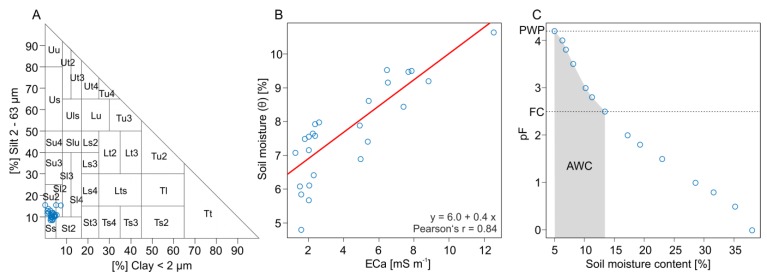
(**A**) Soil texture classes of the 25 lab-analysed reference soil samples. (**B**) Linear regression analysis of apparent electrical conductivity (deep) and soil moisture. (**C**) Mean water retention curve of Königsfeld based on soil texture of the samples and the soil physical data base of [37]. (PWP: Permanent wilting point, FC: Field capacity, AWC: Available water content).

As statistical analyses often show correlations between ECa and clay or sand content, geoelectrical methods are commonly accepted for the estimation of the soil texture [28,51,52]. However, at Königsfeld, the analysis of correlation between ECa_deep_ and soil texture of 25 lab-analyzed reference soil samples showed only poor results. Pearson coefficients of −0.45, 0.43, and 0.25 were obtained for the sand, silt, and clay fraction, respectively. The observation of sometimes weak and inconsistent relationships between ECa and soil properties were also reported by Corwin and Lesch [53] and Sudduth et al. [54]. One reason is that ECa is a cumulative parameter which is affected by a series of soil characteristics such as clay content, soil moisture, salinity, temperature, bulk density, or organic matter content [53,55]. If these parameters are not correlated and if a single parameter is not dominating the influence on ECa statistical analysis becomes difficult. A low range of variation in the control variable can be another reason for poor correlations. At Königsfeld, soil texture varied only within three soil texture classes, namely slightly silty sand (Su2), slightly loamy sand (Sl2), and pure sand (Ss) (Figure 9A). However, the correlation between ECa_deep_ and soil moisture (θ) showed much better results, receiving a Pearson’s r of 0.84 (Figure 9B). Thus, in the next step, the ECa_deep_ data were calibrated on the soil moisture following Equation (4):

(4) $$\theta \;\left[\%\right]=6.0+0.4\;ECa_{deep}$$

where θ is the soil moisture in % and ECa_deep_ the sensor-based apparent electrical conductivity in mS m^−1^ at a depth of 0.37 m. Similarly, Cousin et al. [56] and Besson et al. [57] used the relationship between geoelectrical methods and water content to map the spatial distribution of soil water content at the field scale.

Using the calibration model in Equation (4), soil moisture optimum of the alfalfa-grass mixture was derived from the ECa_deep_ and biomass data. It ranges between 7% and 13%. To better understand the ecological significance and plant availability of the two soil moisture values, we calculated a mean water retention curve on the basis of the mean soil texture data of Königsfeld and the soil physical data base of Wessolek et al. [58] (Figure 9C). Special emphasis was put on the two ecologically important soil moisture states field capacity (FC, pF 2.5) and permanent wilting point (PWP, pF 4.2). FC is the maximum amount of soil water that a soil is able to hold in the root zone against gravity at a matrix potential of −0.33 kPa (pF 2.5). In contrast, the PWP is the amount of soil water that is so strongly retained by the smallest soil pores at a matrix potential of −15 kPa (pF 4.2) [59]. As a consequence, plants are not able to absorb that water and start to wilt. The difference between FC and PWP describes the plants available water content of a soil (AWC) [60]. According to Figure 9C the PWP and FC at Königsfeld is at a soil moisture content of 5% and 13%, respectively. At low soil moistures, biomass production of the alfalfa-grass mixture is already decreasing at soil moistures below 7% even though it is still within the range of the usable field capacity of the soil. This is probably caused by an uneven soil water distribution within the range of AWC especially at low moisture contents [61]. Moreover, plant roots are not uniformly distributed in the soil and the soil water potential that can be overcome by roots differ with different plant species [62]. To increase the water holding capacity (WHC) of these sandy and dry sites, showing ECa below 3.5 mS m^−1^, the farmer can try to ameliorate by spreading organic fertilizers. However, a significant improvement of WHC will require huge amounts of organic matter. Alternatively, an adaptive strategy could include the seeding of grassland species that are more drought-tolerant. 

At high soil moistures, on the other hand, grassland yield begins to decline when soil moisture exceeds field capacity (>13%). Below a pF value of 2.5, water is not held by the soil matrix potential anymore. Instead, it starts to slowly percolate through the soil following the gravitational potential and may contribute to the groundwater below the root zone or is retained by soil layers with poor drainage in the root zone. The latter situation creates water logging with adverse effects on crop growth due to the lack of oxygen. High ECa values were observed at elevated areas, in particular top-slopes, of the field. We assume that at these terrain positions soils were eroded and glacial until it can be found in shallow depths. This is in line with information from the national soil survey [63].

## 4. Conclusions

A grassland field on sandy soils in Northeast Germany was mapped by proximal soil sensors and remote sensing. The UAV-based orthophoto was used to estimate spatial variation in grassland biomass. Soil pH value and apparent electrical conductivity (ECa) from proximal sensing were investigated as indicators for soil-borne causes of biomass variation. The spatial variability and correlation between pH, ECa, and grassland biomass was analyzed by means of semivariance modelling and boundary-line analysis focusing on the yield-limiting behavior of pH and ECa. 

Area-wide mapping of biomass provided means to obtain a sound description of spatial variability by variography based on a large data set. However, the RGB imagery alone does not explain the reasons of spatial variation in biomass. Knowledge about cause-effect relationships is fundamental for taking the appropriate measures in grassland management. Proximal soil sensing in conjunction with boundary line analysis helped to understand some of the drivers of biomass variability. 

Comparing the two proximal soil sensors, the pH meter offers the benefit of direct assessment of a key soil fertility factor. The agronomic interpretation of pH data is straight forward and many recommendation tables exist that relate pH values to decisions on liming or selection of crops or varieties. In contrast, the ECa values are affected by several soil properties, including water, salinity, texture, temperature, and compaction. It requires additional efforts to interpret the ECa data and to transfer them into information that is meaningful in agricultural decision making. Technically, however, the EC sensor showed a better performance than the pH system. The ECa data were less noisy (as derived from the nugget effect parameter in the variogram model), they provided a high spatial resolution due to the higher measurement frequency (1 Hz), and the coulter electrodes did not seriously harm the turf. The marks of the pH sampler, however, were visible even after one year. This can promote the expansion of unwanted plant species in the grassland or initiate soil erosion. Compared to the ECa sensor, the pH data were relatively noisy and much sparser. To improve the pH measurements, better solutions for sampling and/or sample preparation have to be found and measurement time has to be reduced. 

As one of our reviewers pointed out, this study has neglected the temporal aspect of vegetation dynamics. In particular, seasonal variation of plant growth, due to changes in temperature and photoperiod, could modify the spatial distribution of biomass and probably even vegetation composition. For example, above ground plant parts can die off due to frost over winter. When the plants regrow in the following spring, spatial variation of biomass might not be very strong or may even show different spatial patterns over time. Thus, the relationship between biomass and soil properties (ECa and pH) could change within the season. On the other hand, spatial patterns of vegetation composition and biomass might evolve over several vegetation periods. The vegetation might be spatially more uniform after seeding while more pronounced patterns will appear in subsequent years. This evolution of stable and pronounced spatial patterns can not only be attributed to abiotic environmental factors but also to interspecies competition and to species persistence due to accumulation of root systems and/or seeds of certain plant species at certain spots. Unfortunately, we had not enough resources to thoroughly monitor temporal dynamics of spatial vegetation patterns over time in this study. From qualitative observation (field scouting) we had the impression that the patterns of Königsfeld were relatively stable five years after sowing. With regard to the interaction of spatial and temporal dynamics of alfalfa-grass mixtures we suggest that biomass should be assessed a few years after sowing and preferentially later in the vegetation period. However, further studies are required to substantiate this assumption.

## Figures and Tables

**Table 1:** Descriptive statistics of the sensor and laboratory data.

	Minimum	Mean	Median	Maximum	Standard Deviation	Coefficient of Variation
Veris ECa_deep_ [mS m^−1^]	0.5	4.0	3.3	23.8	2.4	0.6
Veris pH	4.9	6.0	6.0	6.9	0.5	0.1
NDVI	0.08	0.23	0.23	0.29	0.02	0.09
Fresh biomass [g m^−2^]	219.1	595.7	678.1	1114.9	308.4	0.5
Dry biomass [g m^−2^]	71.2	146.2	148.3	251.2	51.0	0.3
Clay [%]	0.0	3.3	3.0	7.0	1.6	0.5
Silt [%]	8.0	11.3	11.0	16.0	2.1	0.2
Sand [%]	77.0	85.3	86.0	89.0	2.6	0.03
Soil moisture [%]	4.8	8.1	7.9	11.7	1.3	0.2
